# Comparative Immunogenicities of Full-Length *Plasmodium falciparum* Merozoite Surface Protein 3 and a 24-Kilodalton N-Terminal Fragment

**DOI:** 10.1186/1475-2875-11-S1-P117

**Published:** 2012-10-15

**Authors:** Marvam Imam, Yengkhom Sangeeta Devi, Akhilesh K Verma, Virander Singh Chauhan

**Affiliations:** 1 International Centre for Genetic Engineering and Biotechnology, Aruna Asaf Ali Marg, New Delhi 110067, India, 1 and Synthetic Organic Chemistry, Department of Chemistry, University of Delhi, New Delhi 110007, India

## Background

Merozoite surface protein 3, a leading malaria vaccine candidate, is expressed in schizont stage as 48 kDa non GPI anchored protein which remains associated with merozoite surface after egress. Role of MSP3 in parasite growth is not well defined.-MSP3 is abundantly expressed on the surface of merozoites and is released as a soluble protein. Recently suggested nomenclature has placed MSP3 in a new MSP3 multigene family and termed it MSP3.1. MSP3.1 has been shown to be the least cross-reactive among the members of the MSP3 family. Affinity-purified MSP3 antibodies from the sera of *P*. *falciparum-*exposed individuals and antibodies from mice vaccinated with MSP3 peptides exhibited ADCI activity (15, 21). *Aotus* monkeys vaccinated with yeast (*Pichia pastoris*) expressed full-length MSP3 (MSP3F) were partially protected from a challenge with *P. falciparum* parasites. Antigenicity and functional assays have identified a 70-amino-acid conserved domain in the N-terminal region of MSP3 to be a target of biologically active antibodies. Long synthetic peptides based on the conserved N-terminal sequences, including the 70-amino-acid sequence, have been developed for vaccine trials in humans.

## Materials and methods

Recombinant *Plasmodium falciparum* merozoite surface protein 3 (PfMSP3F) and a 24-kDa fragment from its N terminus (MSP3N) that includes the essential conserved domain, which elicits the maximum antibody (Ab)-dependent cellular inhibition (ADCI), were expressed as soluble proteins in *Escherichia coli.* Both proteins were found to be stable in both soluble and lyophilized forms. Immunization with MSP3F and MSP3N formulated separately with two human-compatible adjuvants, aluminum hydroxide (Alhydrogel) and Montanide ISA 720, produced significant antibody responses in mice and rabbits. Polyclonal Abs against both antigens recognized native MSP3 in the parasite lysate. These two Abs also recognized two synthetic peptides, previously characterized to possess B cell epitopes from the N-terminal region. Antibody depletion assay showed that most of the IgG response is directed toward the N-terminal region of the full protein. Anti-MSP3F and anti-MSP3N rabbit antibodies did not inhibit merozoite invasion or intraerythrocytic development but significantly reduced parasitemia in the presence of human monocytes. The ADCI demonstrated by anti-MSP3N antibodies was comparable to that exhibited by anti-MSP3F antibodies (both generated in rabbit).

## Conclusions

Our work in this study has demonstrated that although immunizations with the full-length MSP3 leads to a substantial antibody response to the epitopes present in the N-terminal region, the N-terminal polypeptide fragment itself elicits a strong and effective immune response that makes it a strong vaccine candidate.

